# Abundance, behavior and entomological inoculation rates of anthropophilic anophelines from a primary Colombian malaria endemic area

**DOI:** 10.1186/1756-3305-6-61

**Published:** 2013-03-07

**Authors:** Nelson Naranjo-Diaz, Doris A Rosero, Guillermo Rua-Uribe, Shirley Luckhart, Margarita M Correa

**Affiliations:** 1 Grupo de Microbiología Molecular. Escuela de Microbiología, Universidad de Antioquia, Medellín, Colombia; 2 Grupo de Entomología Médica. Facultad de Medicina, Universidad de Antioquia, Medellín, Colombia; 3 Department of Medical Microbiology and Immunology, University of California, Davis, CL, USA

**Keywords:** Malaria vectors, Infection rate, Human biting rate, Entomological inoculation rate, Colombia

## Abstract

**Background:**

In Colombia for several years, the Urabá-Bajo Cauca and Alto Sinú region has registered the highest numbers of malaria cases in the country. Malaria vector incrimination and the characterization of entomological parameters will allow for a better understanding of malaria transmission dynamics and the design of effective vector control strategies for this region.

**Methods:**

We conducted a longitudinal survey between November 2008 and June 2010 to quantify entomological (abundance and biting activity) and transmission parameters, including infection rate (IR) and entomological inoculation rate (EIR), to incriminate potential anopheline vectors in three localities of a major Colombian malaria endemic region, the Urabá-Bajo Cauca and Alto Sinú: La Capilla, Juan Jose and El Loro.

**Results:**

A total of 5,316 anopheline mosquitoes corresponding to seven species were collected. *Anopheles nuneztovari* (69.5%) and *Anopheles darlingi* (22.2%) were the most abundant species, followed by *Anopheles pseudopunctipennis* (4.5%), *Anopheles albitarsis* s.l. (2%), *Anopheles triannulatus* lineage Northwest (1.8%), *Anopheles punctimacula* and *Anopheles argyritarsis* (at < 1%, each). Three species were naturally infected with *Plasmodium vivax*, *An. nuneztovari*, *An. darlingi* (IRs < 1%) and *An. triannulatus* (IR = 1.5%). Annual EIRs for these species ranged from 3.5 to 4.8 infective bites per year.

**Conclusions:**

These results indicate that *An. nuneztovari* and *An. darlingi* continue to be the most important malaria vectors in this region. *Anopheles triannulatus*, a species of local importance in other South American countries was found naturally infected with *Plasmodium vivax* VK247; therefore, further work should be directed to understand if this species has a role in malaria transmission in this region.

## Background

Colombia ranks second in number of malaria cases in Latin America and, in the past two decades with few exceptions, more than 100,000 cases were registered annually [1]. For several years, the Urabá-Bajo Cauca and Alto Sinú (UCS) region has had the highest numbers of malaria cases in the country [2], registering 58.18% of the total cases in 2010 [3]. Further, *Plasmodium vivax* has historically been the most prevalent species in UCS, causing 76.11% and 88.26% of the cases in 2010 and 2011, respectively [3,4]. It is likely, however, that these case numbers are an underestimate of both transmission and clinical disease [5,6].

Among the approximately 47 anopheline species that have been identified in Colombia [7], the three main vector species, *Anopheles albimanus* Wiedemann, *Anopheles nuneztovari* Gabaldon and *Anopheles darlingi* Root, are present in UCS, together with local vectors of importance in other Colombian regions including *Anopheles pseudopunctipennis* Theobald, *Anopheles punctimacula* Dyar & Knab, *Anopheles oswaldoi* (Peryassu) and *Anopheles rangeli* Gabaldon [7-10]. Some of these species are sibling species with overlapping characters or belong to a complex of cryptic species that differ in their ability to support parasite development, further complicating their incrimination in transmission [11]. For example, within the Albitarsis Complex, six species have been formally described [12-15] and Ruiz et al. [16] have recently proposed three new members. Within this species complex, only *Anopheles deaneorum* Rosa-Freitas [17-19], *Anopheles marajoara* Galvão & Damasceno [20] and *Anopheles albitarsis E*[21], renamed as *Anopheles janconnae* Wilkerson & Sallum [14], have been described as epidemiologically important in Brazil. In Colombia, a new mtDNA *COI* gene lineage closely related to *Anopheles janconnae* (subsequently named *Anopheles albitarsis* I [16]) was detected in localities of UCS [22], but its importance in malaria transmission is unknown. Similarly, *Anopheles triannulatus* is a complex of at least three species, including *Anopheles triannulatus* s.s. (Neiva & Pinto), *Anopheles halophylus* Silva-do-Nascimento & Lourenço-de-Oliveira, and *Anopheles triannulatus* C (undescribed) [23,24]. In addition, the primary malaria vector *An. nuneztovari* is frequently confused with other species of the Oswaldoi Group due to intraspecific variation and overlapping morphology of the adult [25-28]. Indeed, it has been suggested that *An. nuneztovari* is a species complex [29-32] and based on *white* and *COI* gene sequence analyses, this complex may be composed of at least two cryptic species, *An. nuneztovari* s.s. located in Colombia and Venezuela and *An. goeldii* Rozeboom & Gabaldon and other possible species or lineages present in the Amazon basin [29,31,33]. In Colombia, *An. nuneztovari* was reported infected with *Plasmodium* sp. in Bajo Calima, Buenaventura [34], and more recently, with *Plasmodium vivax* VK247 in Montelibano and Tierralta localities in UCS [8].

In Brazil, *An. triannulatus* at high densities appears to contribute to transmission at the local level [35] and has been reported infected with *Plasmodium falciparum*, *P. vivax* and *Plasmodium malariae* Grassi & Felletiin in the Amazon region [35-37]. This species was also considered the dominant vector in eastern Loreto, Peru [38]. In Colombia, *An. triannulatus* has a wide distribution and in some northwestern localities it was among the predominant species [8], showing either anthropophilic behavior or zoophilic tendencies [8,39]. However, until the present study, *An. triannulatus* had not been reported infected with *Plasmodium* spp. in endemic areas of Colombia.

Previous studies conducted in UCS attempted to increase knowledge of anopheline species behavior [40] and relative importance in malaria transmission [8]. However, these studies were constrained in the number and frequency/seasonality of collection days, which can limit detection of variation in anopheline behavior. Therefore, we conducted a longitudinal survey between November 2008 and June 2010 to assess temporal differences in anopheline behavior and transmission parameters in three UCS localities that have not been evaluated in previous studies.

## Methods

### Study sites

**Figure 1:**
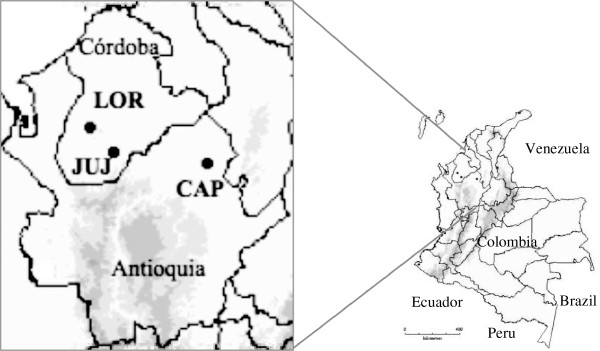
**Collection sites.** UCS region. El Loro-LOR and Juan Jose-JUJ, Córdoba Department and La Capilla-CAP, Antioquia Department.

The sites sampled were (1) El Loro-LOR, in the Tierralta municipality, (2) Juan Jose-JUJ in Puerto Libertador, Cordoba Department, and (3) La Capilla-CAP, in El Bagre municipality, Antioquia Department (Figure 1, Table 1). In general, the primary economic activities in UCS are small-scale agriculture and livestock production. Activities in LOR and CAP sampled sites also include timber extraction and artisanal open sky gold mining, respectively.

**Table 1:** Data on abundance HBR, IR and EIR for the anopheline species collected

**Department/Municipality/Locality**	**Year Month(Number of days)**	**Species**	**N (%)**	**HBR**	**IR% (CI)**	**Annual EIR**
**Antioquia**	**2009 January (6)**	***An. darlingi***	358 (63.9)	14.1	*An. darlingi*	3.7
El Bagre		***An. nuneztovari***	155 (27.7)	6.1	0.087 *Pv* VK210 ^*a*^	
La Capilla-CAP		*An. albitarsis* s.l.	33 (5.9)	1.4	(0.002-0.485)	
07°35^′^N°49^′^W		***An. triannulatus***	8 (1.4)	0.3		
		*An. punctimacula*	6 (1.1)	0,3	*An. nuneztovari* s.s.	3.5
	**2009** April (2), May (4)	*An. nuneztovari*	452 (51.7)	16.7	0.101 *Pv* VK247 ^*a*^	
		*An. darlingi*	366 (41.9)	14.5	(0.003-0.559)	
		*An. albitarsis* s.l.	41 (4.7)	1.7		
		*An. triannulatus*	13 (1.5)	0.5	*An. triannulatus*	4.8
		*An. pseudopunctipennis*	2 (0.2)	0.08	1.515 *Pv* VK247 ^*a*^	
	**2009** August (6)	*An. nuneztovari*	213 (56.5)	8.04	(0.038-8.155)	
		*An. darlingi*	94 (24.9)	3.6		
		*An. triannulatus*	49 (13)	2		
		*An. albitarsis* s.l.	21 (5.6)	0.9		
	**2009** December (5)	*An. darlingi*	329 (62.5)	15.1		
		*An. nuneztovari*	175 (33.3)	7.9		
		*An. triannulatus*	12 (2.3)	0.6		
		*An. albitarsis* s.l.	10 (1.9)	0.5		
**Córdoba**	**2008** November (6)	*An. nuneztovari*	281 (98.9)	6.9		
Tierralta		*An. triannulatus*	2 (0.7)	0.1		
El Loro-LOR		*An. darlingi*	1 (0.4)	0.03		
08°01^′^N 6°07^′^W	**2009** March (6)	*An. nuneztovari*	43 (86)	1.3		
		*An. darlingi*	4 (8)	0.1		
		*An. triannulatus*	3 (6)	0.1		
	**2009** June (6)	*An. nuneztovari*	166 (99.4)	5.3		
		*An. triannulatus*	1 (0.6)	0.04		
	**2009** September (6)	*An. nuneztovari*	64 (100)	2.6		
**Córdoba**	**2009** July (1), Aug (5)	*An. nuneztovari*	1,746 (99.3)	69		
Puerto Libertador		*An. darlingi*	13 (0.7)	0.5		
Juan Jose-JUJ	**2009 November (6)**	***An. nuneztovari***	115 (99.1)	4.4	*An. nuneztovari*	3.6
07°43^′^N 75°51^′^W		*An. darlingi*	1 (0.9)	0.04	0.047 *Pv* VK247 ^*b*^	
	**2010** February (6)	*An. pseudopunctipennis*	236 (62.6)	9.6	(0.001-0.260)	
		*An. nuneztovari*	130 (34.5)	4.6		
		*An. darlingi*	10 (2.6)	0.38		
		*An. punctimacula*	1 (0.3)	0.04		
	**2010** June (6)	*An. nuneztovari*	152 (93.8)	5.2		
		*An. triannulatus*	6 (3.7)	0.3		
		*An. darlingi*	3 (1.9)	0.08		
		*An. argyritarsis*	1 (0.6)	0.04		

*N*: Total number of anophelines collected by period. *HBR*: human biting rate per species (Average of mosquito bites/person/night calculated for each site and collection). *IR*: Infection rate (No. of positive/no. of total analyzed) × 100, ^a^determined by a positive result on the first ELISA carried out with mosquito pools and by nested PCR of individual abdomens of positive pools, ^*b*^determined by the first and second positive ELISAs and by a nested PCR. *CI*: IR confidence interval. *Pv*: *Plasmodium vivax*, *Pf*: *Plasmodium falciparum*. *EIR*: Entomological inoculation rate or the number of potential infective mosquito bites per species per year. Boldfaced: collection period and name of the species with infected mosquitoes. *Anopheles triannulatus* specimens correspond to lineage Northwest [41].

### Mosquito collection

Collections were conducted from November 2008 to June 2010 using human-landing catches, under an informed consent agreement and collection protocol reviewed and approved by a University of Antioquia Institutional Review Board (Comité de Bioética Sede Investigación Universitaria, CBEIH-SIU, UdeA, approval document 07-41-082). Each locality was visited four times, once every three months. Indoor and outdoor collections within ~10 m of the house were conducted regularly by four human baits per shift (two indoors and two outdoors), from 18:00–24:00 h during five days and one additional night from 18:00–06:00 h. For some species it was possible to obtain and rear field-collected larvae to support species identification. Adult mosquitoes and immature stages were identified using morphology based keys [7,42,43]. Species presenting difficulties during the taxonomic identification were confirmed by PCR-RFLP-ITS2 [25,27,44] and *COI* barcode strategy [45] using primers of Folmer et al. [46].

### Detection of *Plasmodium* infected mosquitoes

Enzyme-linked immunosorbent assay (ELISA) was conducted with pools of up to five heads and thorax of mosquitoes of the same species to ensure 99% confidence of detecting at least one infected mosquito per pool (expected < 2%) [8,39]. The initial ELISA was conducted using three monoclonal antibodies directed to *P. falciparum*, *P. vivax* VK247 and VK210, tested on separate plates [8,39,47,48]. Positive pools were confirmed by a second ELISA and a nested PCR with *Plasmodium* genus-specific primers [8,39,49] using 6 μL of DNA extracted from individual abdomens as the template [50]. The PCR analysis served to verify the infected mosquito(s) in the pool.

### Entomological parameters

The infection rate (IR) was calculated as the percentage of *Plasmodium* positive mosquitoes out of the total number of mosquitoes analyzed by species, locality and region. IR values were calculated in two ways: (1) by collection period and (2) for the total number of days sampled. Confidence intervals (CI, 95%) were calculated under the assumption of a binomial distribution using the EPIDAT program, version 3.1 (OPS/OMS 2006). For each site, the summed hourly data of four collections was used to calculate the human biting rate (HBR) as the total number of anophelines captured in each collection divided by the total number of collection days and the average number of collectors [23,51]. To detect variation resulting from differences in mosquito abundances and infection, the annual entomological inoculation rate (EIR) or the number of infective mosquito bites per year per locality was calculated. The annual EIR was obtained by multiplying the average HBR by the number of infected specimens during the four collections per site by 365 days (EIR = HBR × IR × 365).

### Rainfall data

Monthly rainfall data from pluviometric stations situated in proximity to the localities were obtained from the Instituto Colombiano de Hidrología, Meteorología y Estudios Ambientales (IDEAM). Spearman’s correlation was performed to estimate the relationship between rainfall and mosquito abundance using pluviometric data of the previous month to a collection. The analysis was performed using the SPSS Program version 18 (SPSS Inc., Chicago, IL).

## Results

### Anopheline abundance, distribution and seasonal variation

A total of 5,316 anopheline mosquitoes corresponding to seven species were collected during 489 h of sampling (Table 1). CAP and JUJ were the sites showing the highest anopheline diversity with six species each, while in LOR only three species were detected (Table 1). The specimens *An. nuneztovari* were confirmed as such by PCR-RFLP-ITS2. In a *COI* network, grouped with sequences of the subclade IIC of Scarpassa & Conn [33], designated as *An. nuneztovari* s.s. *Anopheles nuneztovari* and *An. darlingi* were the most abundant species (69.5% and 22.2%, respectively) and were present in all three localities. The remaining species found in lower abundances included *An. pseudopunctipennis* (4.5%), *An. albitarsis* s.l. (2%), *An. triannulatus* lineage Northwest (NW) [41] (1.8%), *An. punctimacula* and *Anopheles argyritarsis* Robineau-Desvoidy (each at ≤ 1%). Given that *An. punctimacula* belongs to the Punctimacula Group, which is characterized by a high degree of isomorphism among its species [52-54] these specimens were confirmed using the barcode strategy. The total number of anophelines for each of the species collected did not show a normal distribution (Kolmogorov-Smirnov *Z* = 7.2, *p* < 0.001). The number of collected mosquitoes per night by species varied markedly. For example, specimens of *An. nuneztovari* in LOR ranged from 1 to 55 per night (Mean = 19.6, SD ± 16.7), 4 to 353 per night (Mean = 83.5, SD ± 118.3) in JUJ and 11 to 120 per night (Mean = 39.1, SD ± 24) in CAP. Similarly, specimens of *An. darlingi* ranged from 0 to 5 per night (Mean = 1 SD ± 1.4) in JUJ and 10 to 194 per night (Mean = 46.7, SD ± 40.6) in CAP.

**Figure 2:**
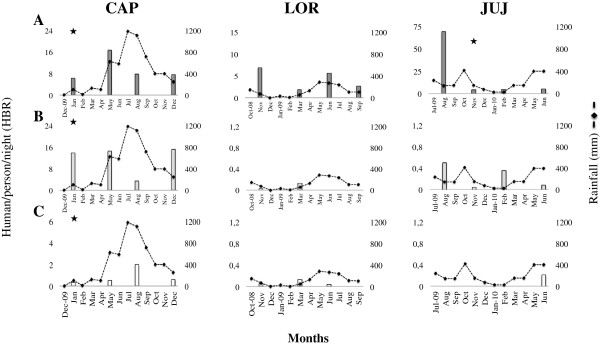
**Relative anopheline abundance in relation to rainfall.** La Capilla-CAP, El Loro-LOR and Juan Jose-JUJ. **A**. *An. nuneztovari*, **B**. *An. darlingi*, **C**. *An. triannulatus* lineage NW [41], *Periods with infected anophelines.

*Anopheles nuneztovari* in CAP was the most abundant species in the second and third collections (April-May and August 2009), accounting for 51.7% and 56.5% of total collections, respectively (Table 1). However in this locality, the peak abundance for this species was observed in the April-May 2009 collection, which coincides with the beginning of the rainy season (Figure 2A). In LOR and JUJ, *An. nuneztovari* predominated in all sites and collections, except in the third collection in JUJ (February 2010), a period of low rainfall when *An. pseudopunctipennis* predominated (62.6%). In JUJ the number of the *An. nuneztovari* was relatively stable for the duration of our collections except for the first collection (July-August 2009) (Table 1), which likely represents an increase related to decreased rainfall (Figure 2A). In LOR, *An. nuneztovari* predominated and, in most collections, accounted for a relative abundance close to 99% and was the only species collected in the fourth sampling (September 2009) (Table 1). The peak abundance for this species in LOR was in November 2008, a period of decreased rainfall. A second peak was observed during the rainy period in June 2009 (Figure 2A).

*Anopheles darlingi* was present in all localities but predominated in CAP with 49.1% of the total anophelines collected. In this site, *An. darlingi* was more abundant during the first (63.9%) and fourth (62.5%) collections, corresponding with the onset (January 2009) and decline of the rains (December 2009), respectively. *Anopheles darlingi* was less abundant than *An. nuneztovari* in the other two collections (Table 1) and was least abundant in the third collection (August 2009), during the rainiest period (Figure 2B). In LOR, *An. darlingi* was present in low abundance in the first and second collections (November 2008 and March 2009), with 0.4% and 8%, respectively. In JUJ, *An. darlingi* was present in low abundances in all collections (0.7-2.6%) (Table 1). In both LOR and JUJ, increased *An. darlingi* densities were temporally associated with the transition periods at the beginning or end of the rains (Figure 2B).

*Anopheles triannulatus* was present in low abundance in all localities. In CAP, peak abundance of *An. triannulatus* occurred in the third collection (August 2009), which coincided with the peak of the rainy season (13%) (Figure 2C). In LOR, peak abundance of *An. triannulatus* (6%) occurred at the beginning of the rainy season (March 2009), and it was not detected in the last collection (September 2009) (Table 1 and Figure 2C). In JUJ, *An. triannulatus* was only collected in the fourth sampling (June 2010), corresponding to a rainy period, with a relative abundance of 3.7% (Table 1 and Figure 2C).

*Anopheles albitarsis* s.l. Galvão & Damasceno was only collected in CAP, present in all collections, with densities ranging from 1.9% to 5.9% (Table 1), with peak abundance at the onset of the rainy season (not shown). *Anopheles pseudopunctipennis* and *An. punctimacula*, considered secondary vectors in Colombia, were detected in CAP and JUJ, in only one collection and in low frequencies (Table 1), during periods of low rain intensity or at the beginning of the rain (not shown). Remarkably, in JUJ, *An. pseudopunctipennis* predominated (62.6%) in the only collection period in which this species was detected, which corresponded to a dry period. Only one *An. argyritarsis* specimen (0.6%) was collected in JUJ during a rainy period (June 2010) (Table 1). There was not a significant (*p* > 0.05) correlation between mosquito abundance and rain for any site or species.

### Biting activity

**Figure 3:**
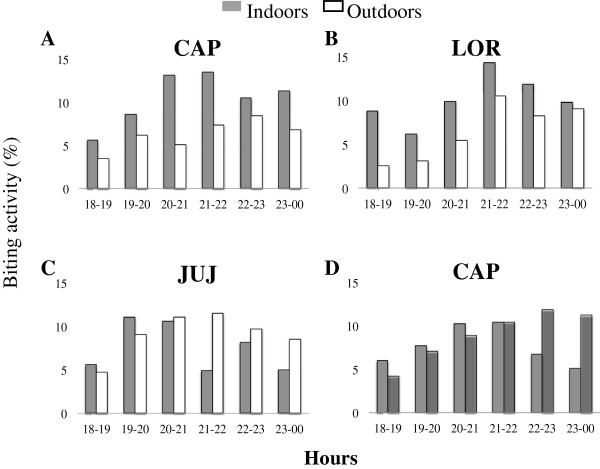
**Mosquito biting activity by hours. A**. *An. nuneztovari* in La Capilla-CAP, **B**. *An. nuneztovari* in El Loro-LOR, **C**. *An. nuneztovari* in Juan Jose-JUJ, **D**. *An. darlingi* in La Capilla-CAP.

Among the species collected that have been identified as potential human plasmodium vectors, none exhibited an exclusive tendency for biting indoors or outdoors and their activity varied in the different localities (Figure 3). In general, 54% *An. nuneztovari* specimens were collected indoors and 46.6% outdoors, values which were not significantly different (*t* = 1.18, *p* > 0.05, n = 71). However, *An. nuneztovari* exhibited a significant difference in indoor/outdoor biting tendency among localities, with endophagic preferences in LOR and CAP (*t* = 2.27, *p* < 0.05, n = 24 and *t* = 2.58, *p* < 0.05, n = 23, respectively) (Figure 3A and 3B). *Anopheles darlingi* did not show marked indoor/outdoor biting preference, with 50.9% of specimens collected outdoors and 49.1% indoors in CAP (Figure 3D). Because of the low number of *An. darlingi* collected in LOR and JUJ it was not possible to analyze biting preferences for this species in these localities.

Mosquito biting activity was determined for the most abundant species from 18:00–24:00 h and during one night from 18:00–6:00 h, and expressed as the mean proportion of mosquitoes collected per hour per species (Figure 3). In general, *An. nuneztovari* exhibited biting activity throughout the night. However, biting peaks varied slightly at collection sites. For example, the highest biting activity was between 20:00–22:00 h, indoors in CAP, and outdoors in JUJ, and between 21:00–22:00 h, indoors in LOR (Figure 3A, 3B and 3C). Biting peaks during the overnight collection were low and occurred at 02:00–03:00 h in LOR, 24:00–01:00 h in JUJ and 01:00–02:00 h CAP (data not shown). The highest biting activity for *An. darlingi* in CAP was outdoors between 22:00–23:00 h (Figure 3D), with an overnight peak registered between 24:00 and 02:00 h (data not shown).

### Human biting rate

Among all species, the primary vectors *An. nuneztovari* and *An. darlingi* showed the highest HBRs (Table 1). The highest HBR was registered for *An. nuneztovari* in JUJ during the July-August 2009 collection (69 bites per night), while *An. darlingi* showed higher HBRs in CAP in three of the four collections, ranging from 14.1 to 15.1 bites per night (Table 1). Surprisingly, *An. pseudopunctipennis,* generally a low abundance species, exhibited a HBR of 9.6 bites per night in the February 2010 collection in JUJ, which was higher than that of *An. nuneztovari* (4.6 bites per night), in the same period (Table 1).

### Infection rate and entomological inoculation rate

A total of 5,299 were processed for infection analysis, four infected specimens were detected in two of the three localities (Table 1). In CAP, *An. nuneztovari* and *An. triannulatus* were infected with *P. vivax* VK247 (IR of 0.101% and 1.515%, respectively) and one specimen of *An. darlingi* was infected with *P. vivax* VK210 (IR of 0.087%). In JUJ, one *An. nuneztovari* specimen was infected with *P. vivax* VK247 (IR of 0.047%) (Table 1). When the IR was calculated by collection period, *An. triannulatus* had the highest IR (12.5%), which was influenced by its low abundance. In general, however, mean annual EIRs were low, with four infective bites per year (Table 1). The HBRs influenced the EIRs, except for *An. triannulatus* (Table 1). In general, cumulative EIR values indicate higher malaria intensity in CAP (12 infective bites per year) than in JUJ (3.6 infective bites per year).

## Discussion

In the UCS region, 24 anopheline species have been previously registered [7]. Historically, the reported malaria vectors included *An. albimanus*, *An. darlingi* and *An. nuneztovari*[8,9]. In the present study, *An. nuneztovari* was the predominant species in two of the three USC localities evaluated (JUJ and LOR), while in CAP, *An. darlingi* predominated, followed by *An. nuneztovari.* Studies conducted in this region during the last decade reported the presence of these two main Colombian vectors [8,40], but those studies did not included periodic samplings that can reveal fluctuations of these main vectors or other species of local importance.

In this study, *An. darlingi* showed higher abundances during the transition periods (at the beginning or end of the rains), consistent with most reports for this species in localities of other Latin-American countries such as Venezuela, Brazil and Belize [55-57]. Occasionally, however, high densities have been reported in the dry season in some regions of Brazil [23,36]. Possible reasons for higher densities during the transition periods have been related to human activities that may provide larval habitats that persist in the dry season or transitional period [58]. In CAP, where *An. darlingi* predominated, the main economic activities are alluvial mining, followed by livestock and small scale rice production, which provide larval habitats for *An. darlingi* that can wash out during prolonged rain periods [56-60]. Low densities of *An. darlingi* in JUJ and LOR may be related to human activities and temporary larval habitats associated with timber extraction, livestock and crop production, which are clearly distinct from the natural rainforest larval habitats typical for *An. darlingi* such as lagoons, forested river margins and streams covered with vegetation [55,61-63].

The dominance of *An. nuneztovari* in Cordoba may be related to environmental disturbance. This species has been characterized by its adaptability and ability to colonize artificial larval habitats in impacted areas [37,64]. In CAP, open sky mining and in LOR and JUJ, artificial ponds for aquaculture and livestock production with the flooding of pastures during rainy periods, can provide the appropriate larval habitats for *An. nuneztovari*[56]. Association of high densities of *An. nuneztovari* with rainy periods has also been reported for other places of Brazil [56] and Venezuela [65].

The low densities found for *An. triannulatus* may be influenced by the collection method since both zoophilic [39] and anthropophilic activity [8] have been reported for this species in the northwest Colombia. *Anopheles triannulatus* was collected at the beginning or during rainy periods when larval habitats associated with lakes and river margins [66], ditches and open sky mining [8,67,68], would be readily available. Previous studies in Venezuela and Brazil reported higher *An. triannulatus* densities during and at the end of the rains [56,59,65]. Given that *An. triannulatus* comprises a species complex, that in Colombia there is no evidence of the presence of the different species and that two lineages were detected with the NW lineage occurring in the UCS region [41], we suggest that the taxonomic status of these specimens should be clarified to draw accurate inferences about the ecological and behavioral characteristics of this taxon.

*Anopheles albitarsis* s.l. was only collected in CAP and showed its highest peak at the onset of the rainy period. Some species of the Albitarsis Complex, such as *An. marajoara*, have been associated with disturbed environments [20,69] such as mining excavations, with higher peaks in the rainy season [55]. However, a better definition of the Albitarsis Complex species present in Colombia is required for proper interpretation of our findings. In contrast to *An. albitarsis* s.l., *An. punctimacula* and *An. pseudopunctipennis* were collected during dry or very low rain periods. This is consistent with previous reports indicating presence of these two species mostly in the dry period [70-72]. Low densities of *An. punctimacula* in NW Colombia [73] were related to its zoophilic tendency [72,74]; therefore, livestock production in CAP and JUJ may provide an additional feeding resource for these species and human landing catches would underestimate their actual abundance.

In this survey, anopheline biting activity varied. Similar to previous reports outdoors [8,40], the highest biting peaks for *An. nuneztovari* in these localities were in the range of 20:00–22:00 h, with activity in both, indoors and outdoors. In CAP, *An. darlingi* predominated, showing biting activity through the night with the highest biting peak outdoors between 21:00–22:00 h. The main biting peaks for these two vectors took place in hours where the people are in their houses involved in leisure activities. Therefore, vector control strategies such as the use of repellents and the applications of residual insecticides may be directed to reduce human-vector contact.

Total HBRs for *An. nuneztovari* and *An. darlingi* differed by locality. HBRs for *An. nuneztovari* were higher than those previously reported for this species in this region [8], or in the east [69,75] and Pacific region of Colombia [69,75]. In Brazil, low HBR values for *An. nuneztovari* have also been reported [23], but also high or higher than the ones found in this study have been reported (30.3 to 123.7 bites per night) [35]. Because *An. nuneztovari* is a complex of at least two species, one in Colombia and Venezuela and the other in Brazil [31,33], ecological and behavioral differences may influence HBR. For *An. darlingi*, HBRs varied significantly among localities and were similar or lower than those previously recorded in other UCS localities [8], and in eastern Colombia [69]. As in the present study, low HBRs have also been reported in the Brazilian Amazon where *An. darlingi* is an important vector [21,23], however, in this region the highest HBRs have also been registered (53.8 to 837.7 bites per night) [35].

Infected mosquitoes were collected during periods of low rainfall, in agreement with previous work in Colombia that related higher malaria transmission to periods of low rain [76]. The low IRs found for *An. nuneztovari* and *An. darlingi* are consistent with those previously found in other UCS sites [8,39,77], indicating that IRs remain relative stable in these localities. Until now, however, *An. triannulatus* has not been incriminated as a malaria vector in Colombia. In this study one specimen from CAP was found infected in the first ELISA and the nested PCR. Although the IR was higher than for the two main malaria vectors, this value was strongly influenced by the low number of specimens collected and analyzed. Our findings should be interpreted in the context of false positives that have been reported mostly for ELISA [78,79], particularly for anophelines with zoophilic preferences [78]. Most importantly, however, a positive PCR indicates presence of the parasite but not necessarily that the mosquito is infective. We also note that *An. punctimacula* and *An. pseudopunctipennis*, species that have been historically considered of local importance [7,9,39], were generally collected in low numbers and were not infected, so their epidemiological relevance in our study sites could not be confirmed.

The IRs, HBRs and EIRs values for *An. nuneztovari* and *An. darlingi* provide additional evidence of the importance of these species in malaria transmission in the UCS sites. EIR, which reflects transmission intensity [80], was influenced by the HBR for *An. darlingi* and *An. nuneztovari* and by the high IR for *An. triannulatus*. The annual EIR for *An. nuneztovari* in CAP and JUJ is considered high for a non-Amazon region where EIRs as high as 141.25 infective bites per year have been reported in the Amazonian locality of Saõ João [35]. At these UCS sites, the EIRs indicate that a person would receive approximately one infected bite every three months. The annual EIR for *An. darlingi* in CAP is similar to the EIR reported for *An. darlingi* in mining areas of southern Venezuela [81]. However, higher EIRs have been reported in forested areas of other Latin American countries where *An. darlingi* occurs at high densities and is a main vector, for example in French Guyana (14.4 to 27.4 infective bites per year) [82], in Rondõnia State (10 infective bites per year) [83], and in Amapá State localities, Brazilian Amazon (up to 1 infective bite per day) [35]. The low EIR registered for *An. triannulatus* is consistent with values previously reported that suggested that this species in high density had importance in local transmission [35]. Although *An. triannulatus* was found to be infected with *P. vivax*, its importance as a malaria vector in Colombia needs to be clarified.

## Conclusions

In the present study, regular field trips over a nearly two year period were conducted in highly endemic UCS to refine knowledge of the temporal and geographic distributions for species considered potential malaria vectors. In general, the results demonstrated that *An. nuneztovari* and *An. darlingi* continue to be the main malaria vectors in UCS localities. Infected specimens were detected in periods when the HBRs for these species were low, indicating that they maintain malaria transmission even in low densities. Furthermore, information on the EIR, an indicator of transmission intensity, may be used to maximize the resources used for control efforts that according to the results should be applied specially in transition and low rain periods. The EIR values are also useful in future studies directed to evaluate the efficacy of the control measure in this area. Additional studies are recommended to investigate the role of *An. triannulatus* as a local vector in Colombia.

## Competing interests

The authors declare that they have no competing interests.

## Authors’ contributions

NND carried out field and laboratory work, data analysis and interpretation, and manuscript draft. DR performed mosquito infection experiments and data analysis. GRU and SL participated in the design of the study, data analysis and critical revision of the final manuscript. MMC conceived and designed the study, coordinated the research group, participated in data analyses and performed critical revisions of manuscript drafts. All authors read and approved the final manuscript.
